# Hot and Cold Water in Eye Diseases

**Published:** 1904-12

**Authors:** 


### HOT AND COLD WATER IN EYE DISEASES.

Nance states in the *Medical Standard* :

1. Heat and cold are best applied to the eye by moist pads. They are more efficacious when applied in this manner than by means of the coil or bladder, in that their action is more penetrating, and their effect is more germicidal.

2. The application of heat is indicated in degenerative corneal processes—interstitial and phlyctenular keratitis, corneal ulcers, pannus, infected corneal wounds, hyphemia, hypopyon, suppurative panophthalmitis, in iritis and cyclitis, in muscular spasm, and in contusion and ecchymosis of the eyelids ("black-eye") to hasten absorption of extravasated blood.

3. The application should be of the highest temperature the patient can endure, viz., 110° to 115° F.

4. They should be employed for a period of fifteen minutes, and repeated at intervals of two or three hours, for many hours.

5. Cold is indicated in hyperemia and inflammation of the conjunctiva. In purulent conjunctivitis it is the remedy *par excellence.* In traumatisms, especially those of the iris and lens, and in the early treatment of contusion of the lids, its employment is of value.

6. In purulent conjunctivitis iced applications may be continuously used for many hours so long as the cornea remains unimpaired, in which instance they are positively contraindicated.

7. Hot applications greatly assist the rapid absorption of various medicaments employed in ophthalmic practice, and when used for this purpose should immediately precede the installation of such solutions.

